# Narrative Identity Reconstruction as Adaptive Growth During Mental Health Recovery: A Narrative Coaching Boardgame Approach

**DOI:** 10.3389/fpsyg.2019.00994

**Published:** 2019-05-08

**Authors:** Douglas J. R. Kerr, Frank P. Deane, Trevor P. Crowe

**Affiliations:** School of Psychology, University of Wollongong, Wollongong, NSW, Australia

**Keywords:** mental health recovery, complex adaptive system, narrative identity, narrative coaching, serious game (boardgame)

## Abstract

**Objective:**

The purpose of this paper is to construct a conceptual framework for investigating the reconstruction of narrative identity in mental health recovery from a complexity perspective. This conceptual framework provides the foundation for developing a health boardgame to facilitate narrative identity reconstruction.

**Methods:**

A selective integrative review of the theoretical and empirical literature relevant to narrative identity reconstruction in recovery was conducted. Sources included books, dissertations, internet resources, and professional journals.

**Findings:**

The reviewed material provides a conceptual framework that offers an enriched understanding of narrative identity reconstruction in recovery as a process of adaptive growth. It identifies the Hero’s Journey, the life story model of identity (LSMI), and intentional change theory (ITC) as particularly relevant in informing strategies for narrative identity reconstruction. The conceptual framework can be operationalized in a narrative coaching treatment approach using a boardgame.

**Conclusion and Implications for Practice:**

In practice, mental health professionals could use the narrative coaching boardgame to facilitate people’s adaptive change with a focus on building skills to reconstruct their preferred narrative identity and foster hope. Future research should explore what aspects of narrative identity and non-linear dynamic processes of change are most important in people’s recovery narratives and in particular these processes can be assessed in response to the use of the boardgame.

## Introduction

Mental health recovery refers to the idea that people with severe and persistent mental illness can pursue psychological wellbeing beyond the limitations of chronic illness (Anthony, 1993; Rogers et al., 2005; Slade and Longden, 2015). Recovery is comprised of various components and processes such as building hope, taking responsibility, gaining a sense of control in life, and building a positive identity (Andresen et al., 2006). Reconstructing narrative identity, to address the loss of sense-of-self and identity that often occurs in mental illness, is a key task for people in recovery (Wisdom et al., 2008). Narrative identity refers to the internal, evolving life story that individuals construct by integrating stories related to their past, present, and future to provide their lives with unity, meaning, and purpose (Bauer et al., 2008). Narrative identity reconstruction entails the formation of an agentic identity where illness is redefined as only one aspect of a complex, multi-dimensional, evolving self that can intentionally choose to pursue wellbeing in recovery. It is a process of change characterized by personal transformation and adaptive growth (Davidson et al., 2005).

Recovery and the key task of narrative identity reconstruction can be understood from a narrative constructivist perspective. In this view, the individual is a self-in-process, ever-changing and adapting to internal and external environmental demands and storytelling is a fundamental process of human functioning (Bruner, 1987, 1991; Mahoney, 1991). As people evolve, so their stories may evolve and thus their narrative identity is open to change (McAdams, 1985; Ricoeur, 1991). The individual can be viewed as a complex adaptive system (Butz, 1997; Rickles et al., 2007; Pincus et al., 2018). This term refers to the complex non-linear nature of the individual, the adaptive evolutionary manner of personal change, and the interconnectedness of the various parts that comprise the individual as a system (Guastello and Liebovitch, 2009). Recovery processes are by nature non-linear as part of human adaptive growth (Deegan, 2001; Onken et al., 2007; Slade, 2010) and are thus highly amenable to being considered from a complex adaptive system perspective.

Non-linear change in recovery is poorly understood and is a difficult concept to apply in recovery-oriented healthcare. A need exists for novel approaches that focus on investigating those processes (Sturmberg, 2016; Graci et al., 2018). Linking narrative identity reconstruction to the complex processes of adaptation and adaptive growth may be a fruitful approach (Rudnick, 2012). A complexity approach considers the often unpredictable and erratic nature of non-linear change processes in life transitions as normal and natural. It can assist people to understand and harness those processes as part of making transitions, leading to adaptive growth and wellbeing in recovery (Bussolari and Goodell, 2009).

Narrative identity reconstruction as a process of adaptive growth can be understood and facilitated by treatment approaches that are strengths-based and target factors involved in non-linear dynamic functioning (Mobus and Kalton, 2015). One such approach is narrative coaching. Narrative coaching is a person-centered, transformational intervention approach that is often focused on identity. It is practical in orientation and commonly utilizes literary metaphors, models, and tools as means to facilitate personal change (Drake, 2010, 2018). A coaching tool in the form of a serious game (boardgame) may have particular salience for narrative identity reconstruction. The term “serious game” refers to games that, while entertaining, model real-life situations and/or have a useful outcome. They aim to promote learning objectives in an engaging and enjoyable manner (Abt, 2002; Fitzgerald and Kirk, 2013). Boardgames are often narrative in design, use a metaphorical approach (Lelardeux et al., 2013), focus on identity, and allow players to experiment with new ways of responding to challenges and explore possible identities (Treher, 2011). They also have the capacity to operationalize complex concepts in a simple manner (Salen and Zimmerman, 2004; Fullerton, 2018).

The main focus of this paper is narrative identity reconstruction during recovery from severe and persistent mental illness. It outlines a conceptual framework in which theories and models related to recovery as a complex process of adaptive growth are integrated in a narrative coaching treatment approach, using a boardgame as a coaching tool. The conceptual framework is underpinned by the common theme of non-linear phenomena, with close alignment between the theories and models outlined (see Table 1). Theoretical integration is operationalized in the boardgame (see Table 2). Narrative coaching to facilitate narrative identity reconstruction is a novel treatment approach in recovery and is aligned with improving wellbeing in patients with chronic conditions. The narrative coaching approach outlined is transdiagnostic and intended for use across common mental disorders. It is transdiagnostic as it targets people’s style of narrative processing (i.e., narrating and interpreting life experiences) that underlies their personal agency. The treatment aim is to facilitate agentic narrative identity reconstruction aligned with mental health and psychological wellbeing in recovery. This approach is aligned with a key advance in the area of treatment for mental disorders, where transdiagnostic dimensions can be understood and targeted in interventions (Krueger and Eaton, 2015; Eaton, 2017; McGorry et al., 2018).

**Table 1:** Alignment between theories and models in an integrative conceptual framework for narrative identity reconstruction in mental health recovery.

Hero’s journey (mental health recovery metaphorical journey)	Life story model of identity (LSMI) (narrative identity)	Intentional change theory (ICT) (personal change model)
**Conceptualization of self**		
Narrative constructivist complex adaptive system	Narrative constructivist complex adaptive system	Narrative constructivist complex adaptive system
**Structure of narrative identity**		
Story stages and plot-points	Storytelling elements	Sequence of change tasks
**Goal underlying personal change**		
Attain a valued outcome	Attain purpose and meaning	Attain a personal life vision
**Personal change characteristics**		
Internal/external challenges	Competing selves/stories	Internal/external barriers
**Personal change mechanism**		
Using inner attributes	Narrative processing	Mindfulness
**Personal change process**		
Non-linear dynamical	Non-linear dynamical	Non-linear dynamical
**Nature of narrative identity reconstruction**		
Emergence of heroic self	Evolving life story	Emergence of ideal self
**Identity change outcome**		
Transformation of identity	Preferred narrative identity	Realization of ideal self

**Table 2:** An overview of the coaching boardgame designed to facilitate narrative identity reconstruction.

Steps in the game (Hero’s journey storyline)	Challenges at each step (Life story model of identity)	Coaching process (Intentional change theory)
**1 The Call** Protagonist recognizes a pressing life issue that must be faced and decides to embark on a journey to address it.	**Preferred identity** *Narrative identity challenge:* Clarify your journey direction and practice the skills you will use along the way.	**Game preparation** Psychoeducation. Coaching goal chosen. Values clarification. Ideal self conceptualization. Mindfulness skills training.
**2 Threshold** Protagonist leaves his/her comfort zone and engages in the recovery journey.	**Underlying beliefs** *Narrative identity challenge:* Choose beliefs that could best support you on your journey.	**Game play** The game-playing mechanism is a five-step reflexive question sequence protocol used at all narrative identity challenges:
**3. Road of trials** Protagonist is fully engaged in the journey and is tested in the process.	**Dominant attitude/s** *Narrative identity challenge:* Choose what attitude/s could best support you on your journey.	• How would your ideal self address this challenge?
**4. Setback** Protagonist is faced with a significant obstacle that must be overcome to make progress.	**Story turning points** *Narrative identity challenge:* Identify a possible main setback on your journey and consider how you could overcome it.	• How is that different from the way you would currently address this challenge?
**5. Rising action** Protagonist is immersed in the journey and faces many competing demands.	**Managing aspects of self** *Narrative identity challenge:* Identify your life roles and consider how to manage them on your journey.	• What qualities/strengths that you have, could you draw upon to address this challenge?
**6. Climax** Protagonist must overcome his/her main personal limitation to succeed.	**Story high point** *Narrative identity challenge:* Identify your main personal limitation on the journey and consider how to address it.	• What archetypes and qualities/strengths could you draw upon to address this challenge?
**7. The return** Protagonist is changed as a person, and shares his/her learnings with others.	**Personal growth** *Narrative identity challenge:* Reflect on your journey learnings and consider how to use them beyond the game.	• Pause and reflect. Based on the above discussion, what action/s can you take to address the challenge? (i.e., support those beliefs; support those attitudes; overcome that setback; manage your life roles; overcome your personal limitation; use your learnings).

The significance of the paper is that it provides a way of integrating concepts and theories with the common theme of adaptive growth (non-linear phenomena) in narrative identity reconstruction during mental health recovery and, further, creates a framework for practically assisting clients to author their preferred narrative identity. This is important as narrative identity reconstruction is a key task in recovery. It is part of attaining psychological wellbeing, which is linked to improved recovery rates and positive outcomes across a wide range of life domains (e.g., education, employment, relationships, health) (Friedli, 2009). The paper is original in that, first, recovery concepts and theories with the common theme of non-linear phenomena do not appear to have been previously integrated in a conceptual framework, and, second, the use of narrative coaching (with a boardgame coaching tool) to facilitate narrative identity reconstruction is a novel treatment approach to promote wellbeing in recovery. The paper will be of interest to mental health professionals, people in recovery, and researchers. For practice it offers a way for mental health professionals to facilitate their clients’ narrative identity reconstruction in recovery. Future research could focus on further clarifying the most important elements of narrative identity reconstruction and non-linear dynamic processes involved in people’s recovery narratives.

## Mental Health Recovery: a Journey of Adaptive Growth and Transformation

Mental health recovery as the pursuit of wellbeing despite chronic illness is a personal journey of healing and transformation in which the focus is on wellness and the fulfillment of people’s potential rather than the treatment of illness. Recovery can be a journey of self-discovery and personal growth (Slade, 2009; Substance Abuse and Mental Health Services Administration [SAMHSA], 2012). By nature, the journey is multidimensional and non-linear with diverse trajectories and an interplay of complex characteristics as part of human adaptive growth (Deegan, 2001; Slade, 2010). It is an intentional, self-directed, sustained endeavor that builds on hope, personal strengths, and valued goals and is characterized by a growing sense of agency where the individual accepts the limitations of illness and discovers a new world of possibility (Deegan, 1996; Drake and Whitley, 2014). In transformational personal change, the individual shifts from a passive to active sense of self. This often entails a rediscovery of self where the individual develops an enhanced ability to reflect on life experiences, learn from them, and take novel action. This is an adaptive process and is considered the essence of recovery (Glover, 2012). Recovery is aligned with a constructivist epistemological perspective. This approach prioritizes subjectivity, the non-linear dynamic processes inherent in personal change, transformation of the self, and the fulfillment of personal potential (Mahoney, 1991; Mahoney and Granvold, 2005; Slade, 2012).

Recovery is narrative in character. Creating individual recovery stories aligned with wellbeing and positive identity is central to mental health recovery (Nurser et al., 2018). Authoring a personal recovery story tends to be an empowering and healing experience for the narrator. People in recovery have the power to tell new stories that will help them overcome adversity and move forward in their recovery (Brown and Kandirikirira, 2007). Their stories are often inspirational and serve to inculcate hope and possibility in others for successful recovery (Kirkpatrick, 2008; Shepherd et al., 2008). A common core narrative in people’s recovery stories is the “quest,” which reframes the experience of illness as an opportunity to undergo personal transformation and attain wellbeing through overcoming difficulties and finding renewed purpose and meaning in life (Frank, 1995). This is an ongoing, redemptive journey in which the individual’s life story shifts from one of chronic disability and stagnancy to a much more complex and dynamic life story (Ridgway, 2001; McAdams and McLean, 2013).

The core “quest” narrative is encapsulated in the hero’s journey (Campbell, 1968) literary metaphorical framework often used in recovery (Lamprell and Braithwaite, 2016; Scottish Recovery and Network, 2016; Foundations Recovery Network, 2018). The hero’s journey is an archetypal quest story referring to both males and females in which the individual as story protagonist undertakes a journey to address a pressing life issue, overcomes internal and external challenges along the way, and in doing so potentially undergoes personal transformation including a changed identity from being a victim to that of a hero (Booker, 2006; Williams, 2017). The hero is an ordinary individual, often an underdog, who finds the courage, resilience, and strength to persevere and endure despite obstacles and setbacks (Allison and Goethals, 2014). The hero’s journey epitomizes the idea that challenges routinely arise in people’s lives and the way that they process and respond to those challenges can mean the difference between poorer or better mental health (Robertson and Lawrence, 2015). The person as protagonist on the hero’s journey gains an understanding that challenges in life are to be embraced rather than avoided and that positivity may be found in moments and experiences perceived as negative (Allison and Goethals, 2017). The hero’s journey is a compelling metaphor for recovery as it encapsulates the challenges and tests of fortitude experienced by people on their recovery journey (Watkins, 2007). It places consumers as the leading protagonist in their recovery journey, enabling them to become active agents in their lives and establish new identities (O’Hagan, 2012). The hero’s journey can be used as a narrative coaching or therapy tool that can be easily learnt and may be used as a scaffold for recovery (Hartley, 2010; Robinson, 2010).

The hero’s journey is aligned with the strengths model of mental health care whereby people take personal responsibility for their recovery and draw on their inner resources to effect positive change in their lives (Rapp and Goscha, 2012). It is also aligned with a posttraumatic growth approach whereby people who encounter psychological difficulties following adversity often find inner strengths and abilities previously unknown and experience a positive change in self-concept (Niemeyer, 2004; Tedeschi and Calhoun, 2004).

## Narrative Identity Reconstruction: Multiple Selves, Stories, and Possibilities

Mental illness often results in people experiencing a sense of loss of self that must be addressed for recovery to become possible (Wisdom et al., 2008; Yanos et al., 2010). The task is for individuals to redefine themselves, to reconstruct a preferred identity aligned with mental health and wellbeing (Slade, 2010). In the transformative process of identity reconstruction, the person gradually sheds the old self and embraces an emergent new sense of self characterized by a more stable and positive identity (Deegan, 2001; Wisdom et al., 2008).

Given that recovery and identity might be seen as narrative, the focus is frequently on narrative identity reconstruction (Bianco, 2011; Nurser et al., 2018). Severe mental illness often drastically diminishes people’s ability to narrate their life story (Gallagher, 2003). Crises of identity, experienced as trauma and personal loss, can undermine the sense-of-self by disrupting the patterns of narrative coherence that are central to a person’s self-concept (Mackenzie, 2008). Constructing a meaningful narrative of self and disorder that promotes recovery is a crucial aspect of identity reconstruction. The challenge for people is to tell stories about their lives in which they are a protagonist characterized by empowerment and agency (Lysaker et al., 2001). Narrative identity reconstruction is based on a view of stories as dynamic, ever-changing, and evolving processes. People’s stories are continually being constructed in interaction with others and the world and are thus provisional and open to change and revision (Mackenzie, 2008). This is important because it allows people to intentionally change and evolve their stories in the pursuit of mental health and wellbeing. People’s stories about their lives are a predictor of psychological wellbeing. Narrative identity has incremental validity in research where it has a stronger relationship with mental health than other common predictors (e.g., gender, personality traits, income) (Adler et al., 2016).

One of the most widely used theories of narrative identity is the life story model of identity (LSMI) (McAdams, 1985, 1993, 2001, 2013, 2018). The LSMI views narrative identity as a person’s internalized and evolving life story that is comprised of smaller stories of a person’s experiences in various life domains (e.g., work, health, relationships). These stories intersect and, in turn, are filled with micro-stories of specific events. The individual’s life story is a cognitive script arranged in a temporal sequence complete with setting, characters, plots, scenes, and themes. Thus, it is complex and dynamic, comprised of multiple stories of the self. Narrative identity can be viewed as a personal myth, in which people make sense of themselves and their lives by creating an imaginary heroic story of self. This includes the use of archetypes (e.g., Warrior, Sage), which are universal story characters with attributes (e.g., courage, wisdom) that can be expressed outwardly in a person’s life. The individual can intentionally call upon archetypal inner resources to facilitate the construction of preferred narrative identity.

Higher levels of personal agency (the feeling of being in control of one’s life) in narrative identity are strongly associated with better mental health and psychological wellbeing (Brown, 2008; Adler et al., 2016). For example, Adler (2012) conducted a longitudinal study of 47 adults undergoing therapy in which participants wrote personal narratives and completed mental health assessments over the course of 12 therapy sessions. It was found that the themes of agency in participants’ stories increased over time, that mental health increased, and that agency and mental health were related. Increased agency appeared in participants’ stories before their mental health improved, and this was likened to participants putting put out a new version of themselves and living their way into it. Davidson and Strauss (1992) conducted interviews over 3 years with 66 persons struggling to recover from prolonged psychiatric disorders. It was found that the reconstruction of an enduring sense of self as an active, dynamical, and responsible agent provides an important aspect of improvement. Identity reconstruction was seen as a process involving (a) awareness of a more agentic sense of self, (b) taking stock of one’s strengths and limitations, (c) putting aspects of the self into action, and (d) using this enhanced sense of self as a resource in recovery. Cochran and Laub (1994) conducted an in-depth small-n qualitative study with people who had undergone psychological trauma resulting from injury. Participants’ initially assumed a victimic identity, but during treatment regained an agentic identity. Participants developed an understanding of themselves as active agents in charge of their lives, able to choose goals and actively direct their activities to achieve them. Identity reconstruction was held to be a correlated movement of the progressive construction of a new agentic life story and detachment from the victim story.

Agency enables people to play a part in their own adaptive growth (Bandura, 2001; Little et al., 2006). Agency is linked to the way that people reflect on their actions in their evolving life story and the sense of choice they experience when considering how to respond to life demands (Adler, 2012). This leads to a sense of control in life in which they are more likely to pursue valued goals and outcomes. There is often dramatic insight into the meaning of life and identity, with the person experiencing a transformation in self-awareness and self-understanding (McAdams, 1985).

Agentic narrative identity is comprised of a narrative agentic self within an agentic plot. The narrative agentic self is a protagonist who intentionally sets goals, strives to achieve those goals, overcomes obstacles, and actualizes ideals. The narrative agentic plot is an ongoing composition that shapes the individual’s evolving life-story (Polkinghorne, 1991, 1996; Cochran and Laub, 1994). It is constantly updated as the individual makes decisions and takes actions in response to life demands (Little et al., 2006). Agentic narrative identity can be taught and learned by the use of models focused on adaptive growth. Models provide inspiration and motivation, portraying a path from the confines of what is to the possibilities of what might be. Cochran and Laub (1994) provide a guide for enhancing personal agency in narrative identity, as follows: (i) study an agentic model with which one can identify; (ii) use storytelling, to imaginatively explore and rehearse the possibilities of the model; (iii) learn skills to move from imagination to enactment in real life.

Agentic narrative identity is aligned with the notion of possible selves, a useful approach in recovery where the individual explores alternative future identities and outcomes in life (Markus and Nurius, 1986; Slade, 2009). Desired possible selves (Tse and Zhu, 2013; Bak, 2015) and desired future narratives (MacLeod and Conway, 2007; Sools et al., 2015) are linked to better outcomes in mental health. The possible self is an imaginary conception of the individual’s future self that encompasses cognitive representations of the person’s hopes, fears, and fantasies (Hoyle and Sherrill, 2006; Erikson, 2007; Slade, 2009). A desired possible self is a behavioral blueprint that motivates the individual, guides behavior in relation to desired outcomes in life, and promotes integrated narrative identity (Cross and Markus, 1991; Singer, 2004; Frazier and Hooker, 2006). An agentic possible self is one that intentionally pursues a preferred identity aligned with valued goals and outcomes (Cochran and Laub, 1994). In a narrative constructivist approach to mental health recovery, the possible self is one of a person’s multiple selves and stories (e.g., current, ideal), any of which may be dominant at a given time in a given context (Mahoney, 1991; Mahoney and Granvold, 2005; Bianco, 2011). The possible self must compete with co-existing identities that are mutually reinforcing, in tension, contradictory, and incompatible (Davidson et al., 2005). Multiplicity of selves can reinforce mental health difficulties or contribute to a healthy sense-of-self aligned with mental health and psychological wellbeing (Koch and Shepperd, 2004). Mental health presupposes an integrated narrative identity with a diversity of selves and stories existing in relative harmony and co-operation (McAdams, 1985; Singer, 2004).

Constructing narrative identity as an active process involves the use of narrative processing. This refers to the filtering of life experiences through a template where people perceive, select, and plot their lives using narrative devices such as imagery, characters, plot, goals, and underlying morals or themes (Sarbin, 1986; Singer and Bluck, 2001; Singer, 2004; Riessman, 2008). Autobiographical reasoning is also used and refers to the meaning that people make of their created narratives (Habermas, 2011). The person’s point of view (e.g., agent, victim) in narrative processing is critically important. How the person makes sense of a life experience and acts on it will emerge from that point of view (Park and George, 2013). Optimal mental health and psychological wellbeing are associated with transformational narrative processing where the person openly explores difficult life experiences, finds a positive ending to these challenges, and grows from the experience (Pals and McAdams, 2004). Transformational processing is contrasted with ruminative processing, in which the person is unable to let go of old selves and goals (King, 2001; Pals and McAdams, 2004; Pals, 2006a,b; Whitehead and Bates, 2016).

## The Narrative Constructivist Self in Recovery: Complex Change and Adaptive Growth

The narrative constructivist self in recovery as a complex adaptive system is an open system, intelligent, meaning-making, intentional, proactive, ever-changing, adaptive, and ever-evolving. It is a self-in-process, in a constant state of flux and becoming, underpinned by non-linear dynamical processes of human functioning. The self is inherently growth-seeking and is teleonomic (self-driven) rather than teleological (goal-driven) (Mahoney, 1991; Niemeyer, 1993; Perna and Masterpasqua, 1997; Chamberlain, 1998). Personal growth, development, and transformation are inherent in the change processes of the narrative constructivist self and individuals are viewed as active participants in their own lives (Mahoney and Granvold, 2005). This perspective is a helpful model of self when applied to mental illness since it opens up the possibility of adaptive growth in relation to the challenges inherent in the recovery journey (Slade, 2009).

Adaptive growth as part of personal change in recovery involves both first-order, developmental (gradual) growth and second-order, transformational (abrupt) change (Gelo and Salvatore, 2016). Adaptive growth is constrained or facilitated by people’s potential to respond adequately to internal and/or external challenges (Mahoney and Marquis, 2002). From a complex adaptive systems perspective humans have inherently high levels of adaptive capacity, which allows them to proactively shape their life-course rather than just respond in a reactive manner to challenges. This affords them a sense of personal agency and identity (Little et al., 2006). People can enhance their adaptive capacity by engaging in personal growth exercises such as developing creative flexibility in decision-making and problem-solving (Mahoney and Granvold, 2005; Mobus and Kalton, 2015).

In relation to mental health recovery, psychopathology is a dynamical system state of equilibrium where people’s habitual patterns of functioning interfere with their everyday functioning and undermine wellbeing (Mahoney and Marquis, 2002). System destabilization is a requisite for adaptive growth as the person’s functional pattern will continue unless challenged. For system reorganization to take place, old functional patterns must be altered or replaced. Optimal functioning and better mental health entail a turbulent balance between stability and instability as well as order and disorder in which the person is stable yet flexible and agile, trying novel responses to find the most adaptive system state to meet internal and/or external environmental demands (Salvatore et al., 2015; Gelo and Salvatore, 2016). The main characteristic of adaptive growth in complex adaptive systems is multiplicity of possible outcomes, where an individual can explore and choose behavior in response to demand (Plsek and Greenhalgh, 2001).

Intentional change theory (ICT) (Boyatzis, 2006; Boyatzis and McKee, 2006) is a model for sustainable personal change aligned with the concept of adaptive growth that may also be used to facilitate narrative identity reconstruction. ICT is a self-directed learning framework that uses the lexicon of complex adaptive systems to describe personal change. The goal is for the individual to attain a desired ideal self (e.g., preferred narrative identity) in the context of pursuing an affectively compelling personal life outcome. The ICT change process entails movement through a sequence of five challenge steps in which the person answers a series of questions that, when successfully addressed, facilitates construction of the ideal self. Movement is from a current, undesired state-of-being (current self) which functions as a *negative emotional attractor* (i.e., habitual pattern of functioning) to a desired state-of-being (ideal self) which is a *positive emotional attractor* (i.e., novel pattern of functioning). This is a transformative shift in the individual that may be viewed as second-order change (Gelo and Salvatore, 2016). Mindfulness is viewed as a central change mechanism with the aim of raising a person’s awareness in order to intentionally engage in desired personal change. It is theorized that by increasing people’s understanding of the complex nature of personal change, they learn to harness the processes rather than fear or misunderstand them (Boyatzis and McKee, 2006).

## Narrative Coaching: Agentic Identity in the Making

The shift from a pathology orientation of illness and disability in recovery to a focus on mental health and psychological wellbeing has resulted in the use of positive, strengths-oriented interventions such as narrative coaching (Bora, 2010; Bora et al., 2010; Slade, 2010; Cavanagh and Buckley, 2014). Narrative coaching is aligned with identity reconstruction in that it is an experiential approach that assists people to revise their personal narratives to gain fresh perspectives, pursue novel possibilities, and attain desired outcomes in life. Narrative coaching helps people become more aware of their choices in life which in turn provides an opportunity for them to intentionally author the multiple stories that comprise their narrative identity and help transform their illness narratives into healing ones (Drake, 2010, 2017, 2018).

Transformational identity change in narrative coaching can be facilitated by the use of reflexive questioning which facilitates the person’s ability to think about his or her own belief systems and make new connections. This process focuses on getting a person to investigate their interactions via introspection as they happen (differentiated from reflective thinking, which refers to thinking following action). Reflexivity encourages people to question their attitudes, thought processes, values, assumptions, prejudices and habitual actions, strive to understand their life roles, and appreciate how they influence their actions (Oliver, 2005). Reflexive coaching questions are an essential tool to facilitate self-awareness and assist individuals to reframe difficulties in a novel manner and find solutions to their problems (Hawkins and Smith, 2014).

Narrative coaching often involves the use of coaching tools to facilitate personal transformation (Biswas-Diener, 2010; Boniwell et al., 2014). Serious games are increasingly used in coaching, and this includes the use of boardgames. Their focus on identity and exploring possible identities makes them highly relevant for narrative identity reconstruction in recovery. Agency is a critical factor in boardgames, where players experience choice of response and a sense of control over the game’s outcome (Fullerton, 2018). This allows them to develop new concepts of self and the world and learn new, adaptive skills that they can use in real life (Mitgutsch, 2011). This is part of game-based learning in which the person develops a mental model that matches the game system which, in turn, models a real-world system (Wasserman and Banks, 2017).

## Hero’s Recovery Journey Boardgame: a Crucible for Adaptive Growth

Based on the theoretical and practical models outlined above we have developed a boardgame designed to facilitate people’s narrative identity reconstruction in recovery. The boardgame is designed to be used as a tool as part of narrative coaching. The boardgame (titled, *“Heroes and heroines: The recovery journey boardgame”)* is an immersive role-play experience designed to be a crucible for people’s adaptive growth in recovery. The boardgame integrates game elements that represent the key components of mental health recovery, narrative identity reconstruction, and complex adaptive systems. This encompasses simple rules, board, avatar, game-playing guide, and playing cards that are carefully selected, operationalised, and integrated. ICT is used as the narrative coaching framework, the LSMI (McAdams, 1985) is used to represent narrative identity challenges within the hero’s recovery journey storyline, and a reflexive coaching style (Oliver, 2005) embedded with applied mindfulness skills (Langer, 2000) is used as the game-playing mechanism (method used by player and coach to interact with the game world). The boardgame is based on established principles of game design that includes detailed conceptualization and iterative play-testing (i.e., test, analyze, refine, repeat) followed by a pilot program to ensure the game achieves its intended aim (Salen and Zimmerman, 2004; Adams, 2014; Schell, 2014; Fullerton, 2018).

The boardgame simulates the hero’s journey (Campbell, 1968). It is a model-representation of the hero archetype (agentic self) within a hero’s journey storyline (agentic plot) in which the player as protagonist engages in his or her own hero’s recovery journey in pursuit of a valued real-life goal (personal life vision). The purpose of the game is for the person to shift from an undesired current self narrative identity (ICT *negative emotional attractor*) (i.e., habitual pattern of functioning) to a preferred ideal self narrative identity (ICT *positive emotional attractor*) in relation to the chosen goal. Players traverse a sequential agentic storyline consisting of hero’s journey steps (e.g., Threshold; Road of Trials) by completing narrative identity challenges (e.g., choosing helpful beliefs and attitudes that support goal attainment) that represent important components of narrative identity. Once players complete a narrative identity challenge, they move on to the next storyline step until all the steps in the game are completed. In completing the journey, players construct a preferred narrative identity and potentially experience personal transformation. Players also learn about the complex processes of adaptive change and how they might be harnessed in recovery. Simple metaphors are used to explain the complex non-linear processes involved in personal change. For example, attractors are explained as habitual behavioral routines and are referred to metaphorically as “life-magnets” where the person is “pulled” repeatedly in a given direction, and the aim is to create a new desired ideal self attractor “life-magnet” to replace the current self attractor.

The game-playing mechanism, used iteratively at the narrative identity challenges, is a critical component of the game. Players engage in a coaching question sequence at each step in the game where they consider (1) how they as their ideal self might address the challenge, (2) how that differs from their current self response, (3) what known personal strengths/qualities they could draw upon to meet the challenge, (4) what agentic archetypal inner attributes they can draw upon, and (5) what action they could take to meet the challenge. Players use applied mindfulness skills in the question sequence in which they engage in an adaptive process of experimentation, engaging in novel ways of thinking (i.e., agentic ideal self perspective) to search for the best solutions to address the challenge. Players refer to a set of agentic archetype cards (i.e., Warrior, Sage, Adventurer) to consider which archetypal strengths/qualities they could draw upon to meet the challenge. For example, the player might choose the Warrior to meet a given challenge and must consider which of the related attributes of skill, courage, discipline, and determination might be used. The iterative, reflexive coaching process promotes in-depth consideration of agentic attributes and how they might be used. In considering agentic attributes and experimenting with related agentic responses to the challenges, players engage in transformational narrative processing where they can shift from a victim identity to an agentic identity. Moving from step to step in the game, as narrative identity challenges are completed, players learn an agentic cognitive schema (hero’s journey) and script (personal change process) which is potentially internalized in their narrative identity reconstruction. This is the mindset and cognitive skills of the everyday hero who, above all, has the adaptive capacity to overcome difficulties and attain success on his or her journey. In line with game-based learning, it is envisaged that the transformative nature of the coaching intervention will translate into real-life skills for use beyond the boardgame.

## Conclusion

Using a narrative coaching treatment approach aligned with complex change processes inherent in adaptive growth provides an integrated framework (see Table 1) that may be of value in understanding and facilitating narrative identity reconstruction as part of psychological wellbeing in recovery. The development of a boardgame to facilitate narrative identity reconstruction has several research and practical implications. Future research should explore what aspects of narrative identity and non-linear dynamical processes of change are most important in people’s recovery narratives, with a view to assisting them to strengthen and leverage those aspects of self in constructing a preferred narrative identity. In practice, mental health professionals could use the game to engage their clients in recovery, offer a model of adaptive change that normalizes the often irregular and uncertain journey of recovery, assist clients to build skills to reconstruct their preferred narrative identity, and foster their hope for a journey toward wellbeing and the fulfillment of their potential.

## Author Contributions

All authors listed have made a substantial, direct and intellectual contribution to the work, and approved it for publication.

## Conflict of Interest Statement

The authors declare that the research was conducted in the absence of any commercial or financial relationships that could be construed as a potential conflict of interest.
